# Serum Transcobalamin Concentration in Cats—Method Validation and Evaluation in Chronic Enteropathies and Other Conditions

**DOI:** 10.3390/vetsci11110552

**Published:** 2024-11-09

**Authors:** Tim Kunath, Stefanie Kather, Franziska Dengler, Ebba Nexo, Helga Pfannkuche, Romy M. Heilmann

**Affiliations:** 1 Department for Small Animals, College of Veterinary Medicine, University of Leipzig, 04103 Leipzig, Germany; 2 Institute of Veterinary Physiology, College of Veterinary Medicine, University of Leipzig, 04103 Leipzig, Germany; pfannku@vetmed.uni-leipzig.de; 3 Institute of Physiology, Pathophysiology and Biophysics, University of Veterinary Medicine Vienna, 1210 Vienna, Austria; franziska.dengler@uni-hohenheim.de; 4 Department of Livestock Tissue Metabolism, Institute of Animal Science, University of Hohenheim, 70599 Stuttgart, Germany; 5 Department of Clinical Biochemistry, Aarhus University Hospital, 8200 Aarhus, Denmark; enexo@clin.au.dk

**Keywords:** cobalamin, enzyme-linked immunosorbent assay, feline, folate, haptocorrin, holotranscobalamin, hypercobalaminemia, inflammation, metabolism, neoplasia, vitamin B_12_

## Abstract

Hypocobalaminemia is common in cats with chronic enteropathy (FCE). However, the disruptions in cobalamin metabolism are not fully understood and may vary across species. Cobalamin is distributed to target tissues via binding to transcobalamin (TC) in blood, which has not been evaluated in cats. Thus, an in-house sandwich-ELISA was established to evaluate serum total TC concentrations in cats with FCE. Surplus sera served to analytically validate the assay, and serum TC concentrations were compared among cats with FCE and other diseases (gastrointestinal neoplasia, cholangiohepatopathy, and other neoplastic or non-neoplastic conditions) and healthy controls. Observed-to-expected ratios for serial dilutions ranged from 72.4 to 145.6% and were 75.1–126.7% for spiking-and-recovery. Intra- and inter-assay variability was <17.7% and <17.2% and the preliminary reference interval for feline serum TC was <160–2795 aU/L (lower detection limit: 160 aU/L). Serum TC levels were significantly decreased (*p* = 0.0067) but not correlated with paired cobalamin concentrations in FCE. Hypertranscobalaminemia predominated with hypercobalaminemia, reaching the highest levels in advanced-stage chronic kidney disease (CKD) cases. TC variations in cobalamin deficiency states with FCE may be linked to inflammation or autoantibodies. This and possible links between serum TC variation in FCE, intracellular cobalamin availability, response to supplementation, and concurrent CKD require further exploration.

## Simple Summary

Decreased serum cobalamin (vitamin B_12_) concentrations are common in cats with chronic gastrointestinal disease, but the underlying alterations in the metabolism of cobalamin are currently not exactly known and may differ from those in other species. Cobalamin transport to peripheral tissues requires binding to transcobalamin (TC), but this transport molecule has not been investigated in cats. Thus, an immunoassay to measure TC in serum samples from cats was established, its performance validated, and it was used to measure serum TC concentrations in cats with chronic gastrointestinal disease in comparison to healthy controls and cats with other conditions. Significantly decreased serum TC levels were seen in cats with chronic gastrointestinal disease, but these were not related to the concentrations of cobalamin in serum. Severely increased serum TC concentrations, on the other hand, were frequently detected in cats with increased serum cobalamin levels, particularly cats with advanced chronic kidney disease (CKD). Reduced serum TC levels in cats with chronic gastrointestinal disease could be linked to the inflammatory process or development of autoantibodies. Possible links between serum TC levels and cobalamin availability in tissues, response to supplementation, and CKD need further study.

## 1. Introduction

Feline chronic enteropathy (FCE) is accompanied by chronic gastrointestinal clinical signs, including vomiting, diarrhea, and weight loss of at least 3 weeks’ duration, and requires the exclusion of other underlying diseases (e.g., hyperthyroidism, diabetes mellitus, exocrine pancreatic insufficiency, endoparasites, or other infectious intestinal diseases) [1,2,3]. As a common condition in older cats, its prevalence has increased over the last decade [1,2,3,4,5]. Disease activity in cats with gastrointestinal signs, particularly with suspicion of FCE, is commonly assessed using the feline chronic enteropathy activity index (FCEAI) [6]. This activity index evaluates a combination of clinical signs, laboratory parameters (e.g., total white blood cell count, serum albumin concentration, and alanine aminotransferase activity), and endoscopic observations to assess the severity of the disease [6]. FCE is mostly classified as either chronic inflammatory enteropathy (CIE) or low-grade intestinal lymphoma (LGIL) [2,4,5]. The etiopathogenesis of CIE is incompletely understood. Based on current consensus, it involves a combination of environmental factors, genetic predisposition, and dysregulated immune response [2,5,7]. CIE is currently subclassified by the response to treatment, differentiating food-responsive enteropathy (FRE) from immunosuppressant-responsive enteropathy (IRE) cases. Both entities cannot necessarily be differentiated based on clinical signs, clinicopathologic findings, or histopathology [1,2,5]. Regardless of the underlying cause, 17–78% of cats with FCE have serum cobalamin deficiency [8,9,10].

Cobalamin, also referred to as vitamin B_12_, is a water-soluble vitamin [11,12]. It is mainly ingested via food of animal origin, and a small percentage might also be provided by the intestinal microbiota [11,13]. Following the preparatory phase of digestion in the stomach, cobalamin is bound to haptocorrin at an acidic pH. In the duodenum, pH increases, haptocorrin is degraded, and cobalamin is transferred to intrinsic factor (IF) [11], a vitamin B_12_-binding protein that in the cat is produced almost exclusively in the exocrine portion of the pancreas [11,14,15]. As a next step, the IF–cobalamin complex is absorbed in the ileum via a receptor-mediated process [16,17]. From the intestinal epithelial cells, cobalamin reaches the bloodstream and is transported to the target tissues bound to transport proteins [17,18], which are termed transcobalamin and haptocorrin (previously named transcobalamin 0 and 1) [16,19,20,21,22,23].

Cobalamin plays an important role in cell division and differentiation. It serves as a cofactor of methylmalonyl-CoA mutase, a key enzyme in the citrate cycle, and methionine synthase, an important enzyme in methionine metabolism [11,12,16,24]. The reduced activity of methylmalonyl-CoA mutase in cats leads to an accumulation of methylmalonic acid (MMA) [25]. An undetectable low serum cobalamin concentration, together with an increased MMA concentration, is interpreted as evidence of intracellular cobalamin deficiency [11,25]. This differs from hypocobalaminemia, defined as a serum cobalamin concentration below the reference interval (RI). Currently, cobalamin status in cats is categorized as either normocobalaminemia (corresponding to a serum cobalamin concentration within the RI), hypocobalaminemia, or hypercobalaminemia (serum cobalamin concentrations above RI) [11,12].

Hypocobalaminemia occurs primarily in cats with gastrointestinal diseases and exocrine pancreatic insufficiency. In very rare cases, it can be due to a dietary deficiency [11]. Transient hypocobalaminemia in cats with hyperthyroidism is also described [26,27]. Though serum concentrations of cobalamin do not necessarily reflect the cobalamin status at the cellular level, supplementation is generally recommended for cats with hypocobalaminemia or low-normal serum cobalamin concentrations [28]. Disturbed absorption into the cells, however, can also lead to an intracellular undersupply of cobalamin despite normocobalaminemia. Treatment-naïve hypercobalaminemia (i.e., without prior supplementation of cobalamin) has been primarily associated with hepatic and neoplastic disease in humans and cats [19,29].

Cobalamin metabolism in cats, particularly cats with FCE, remains to be better understood in the context of the pathogenesis of FCE and for diagnostic and therapeutic considerations. In addition to measuring the concentration of cobalamin (separated from its binding proteins), other markers of cobalamin trafficking in the blood would need to be studied and evaluated in relation to the serum cobalamin status. Transcobalamin (TC; previously referred to as transcobalamin II) is a 47 kDa protein with an essential role in delivering cobalamin to cells and tissues [22], and is mainly produced in hepatocytes, but intestinal, endothelial, and monocytic production is also reported [30,31]. TC transports about 20% of the circulating cobalamin in humans [31,32] and about 75% in cats [12,20], but has not been evaluated in association with serum cobalamin status or diseases in cats.

This study aimed to establish and validate an enzyme-linked immunosorbent assay (ELISA) for measuring serum TC in feline serum, and to measure serum TC concentrations in cats with FCE compared to other diseases affecting serum cobalamin status and healthy controls. We hypothesized that cats with FCE, particularly those cats with hypocobalaminemia, (1) have changes in serum TC concentrations as a cause or consequence of the disease, and that (2) serum TC concentrations in FCE are comparable to TC levels in cats with other conditions linked to changes in cobalamin metabolism (e.g., via dysregulation of cobalamin transport proteins or their cellular receptors).

## 2. Materials and Methods

### 2.1. Sampling Population and Sample Collection

Surplus serum samples from 254 cats (*n* = 147 stratified into different groups based on health status or primary disease and *n* = 107 for assay validation) presented at the Department for Small Animals at the University of Leipzig (UL), Germany, were used for the study. These samples were archived (stored at −20 °C for a maximum of 24 months, at which serum TC was shown to be stable long-term in humans [33]) at the Veterinary Diagnostic Laboratory at the Department for Small Animals at UL. Owners of the cats gave their written consent to the use of left-over samples and anonymized patient data by signing the hospital admission form approved by the ethics committee of the UL College of Veterinary Medicine. Thus, ethics approval for the use of these samples was not required. Some of these cats had serum cobalamin measured (Advia Centaur XP, Siemens, Munich, Germany) as part of their routine diagnostic evaluation.

Human serum samples used for the study were archived (surplus) materials obtained from the local blood bank at the College of Medicine at UL, Germany, and had passed all standard pre-donor screening tests.

### 2.2. Western Blot

The specificity of the antibodies raised against recombinant human TC in rabbits (rabbit anti-recombinant human TC antibodies) [34] to detect feline TC was determined prior to cross-validating the TC-ELISA established to measure serum TC concentrations in dogs [35]. The human serum (diluted at 1:100) and three different feline serum samples from cats with chronic gastrointestinal signs (each diluted at 1:10) were subjected to sodium dodecyl sulfate-polyacrylamide gel electrophoresis (SDS-PAGE, Mini-PROTEAN^®^ Tetra Cell, Bio-Rad, Feldkirchen, Germany) and electrotransferred onto a nitrocellulose membrane (Nitropure 0.45 µm, Osmonics, Westborough, MA, USA) using an EasyPhor Semi-Dry Blotter (Biozym, Vienna, Austria). Membranes were preincubated in 2% bovine serum albumin (BSA) in Tris-buffered saline (TBS) containing 0.2% polyoxyethylene-20 sorbitan monolaurate (Tween-20; TBST buffer) for one hour and then incubated overnight under gentle agitation at 4 °C with the primary (anti-TC) antibody (diluted at 1:1000). After washing with TBST, the membranes were incubated for one hour at room temperature under gentle agitation with a horseradish peroxidase (HRP)-coupled secondary (donkey anti-rabbit) antibody (Cellsignalling #7074; 1:10,000). Membranes were then rinsed again with TBST, and the signal indicating protein bands was detected by enhanced chemiluminescence (Clarity Western ECL Substrate, Bio-Rad) using a ChemiDoc IT 600 Imaging System (UVP/Analytik Jena GmbH, Jena, Germany) and analyzed with the VisionWorks^TM^ software v.9.1.20063.7760 (Analytik Jena GmbH).

### 2.3. Sandwich ELISA for Serum Feline TC Measurement

The ELISA procedure was modified from previous protocols [34,35]. Briefly, enhanced-binding ELISA plates (96-well High Binding Microtiter Plates, Greiner Bio-One, Frickenhausen, Germany) were coated with the primary antibody (a γ-globulin fraction of rabbit anti-recombinant human TC at 200 ng/well) in 200 mM carbonate-bicarbonate, pH 9.4 (ThermoFisher Scientific, Dreieich, Germany). After sealing, the plates were incubated overnight at 4 °C and washed three times with 0.02 M Tris/HCl, 0.15 M NaCl, 0.05% (*v*/*v*) Tween-20, pH 7.5 (TBST; wash buffer). Nonspecific binding sites were blocked using 250 μL TBST that was supplemented with 5% (*w*/*v*) BSA (fraction V), pH 8.0 (assay buffer). Plates were then incubated for one hour at 37 °C and washed thrice. Calibrators (human serum with known holotranscobalamin concentration [measured at the MVZ Human Clinical Pathology Laboratory, Leipzig, Germany] that were serially diluted 1:5–1:320 in TBS assay buffer), blanks (assay buffer), and feline test samples (diluted 1:2 in TBS; determined by screening 107 archived serum samples to target the linear range of the assay for most of the measurements) were then applied in duplicates of 100 μL solution each. Because the exact concentration of serum TC remained unknown, the highest calibrator was assigned a value of 18,500 aU/L (arbitrary units per liter). Feline test samples with a TC concentration above the assay calibrator range upon first testing were further 2-fold diluted and re-assayed.

After incubating for two hours at room temperature and another three plate washes, 100 μL of the biotinylated (EZ-Link^®^ Sulfo-NHS-LC-Biotin, ThermoFisher) anti-TC antibody (γ-globulin fraction of rabbit anti-recombinant human TC) in assay buffer was added to each well (30 ng/well). Plates were then incubated at 37 °C for another hour, washed three times with TBS, and received 100 μL NeutrAvidin-HRP each (ThermoFisher; 20 ng/well). Following a last incubation for 30 min at 37 °C, the plates were washed three times and developed after applying a stabilized 3,3′,5,5′-tetramethyl-benzidine substrate solution (Pierce^TM^ TMB Substrate kit, ThermoFisher). Protected from light, the plates were then incubated for six minutes, after which a stop solution (Invitrogen^TM^ ELISA Stop Solution, ThermoFisher) was added, and the plates were gently agitated for a few seconds on a vortex mixer. The absorbance in each well was measured using an automated microplate reader with a filter for 450 nm (EPOCH^TM^ 2 Microplate Spectrophotometer, BioTek, Winooski, VT, USA). The results were analyzed using the Gen5^TM^ v3.10.06 data analysis software (BioTek) and a 5-parameter logistic curve fit (y = *f* [x] = d + [(a–d)/(1 + (x/c)b)e]; y = dependent variable, x = independent variable, and a through e = determinants of the curve shape), with TC concentrations calculated for feline serum samples by transposing the blank-subtracted A_450nm_ obtained for these test samples onto the calibrator curve.

### 2.4. Analytical Validation of the ELISA for Use with Feline Serum

The modified sandwich immunoassay was analytically cross-validated by determining the lower detection limit, dilutional linearity, spike-and-recovery, intra- and inter-assay variation, possible interference with cobalamin binding, and a preliminary reference interval (RI) for feline serum TC [36]. The lower detection limit of the assay (LoD) was calculated as the mean response plus three standard deviations (SD) for 20 replicates of the blank solution (TBST assay buffer) transposed onto the calibration curve, and the lower limit of quantification of the assay (LoQ) was determined by repeating the procedure using a feline serum pool with no detectable TC. Dilutional linearity was determined by evaluating feline serum samples at a serial two-fold dilution from 1:2 to 1:8 for three serum samples and two serum samples with a higher TC concentration at a serial two-fold dilution from 1:2 to 1:16. The remaining validation parameters were determined using either individual feline sera (if large volumes were available after screening for TC concentration) or pooled feline serum samples (combining smaller-volume samples with similar TC concentrations) with low, moderate, or high TC concentrations. Spike-and-recovery was determined by mixing equal volumes of eight different feline sera/serum sample pools to obtain four spiked samples and calculating the percentage of TC recovery for those samples ([observed value (aU/L)/expected value (aU/L)] × 100%) in a xenospecies assay format. The intra-assay variability of the ELISA was assessed by assaying four feline sera/serum sample pools four times each within the same assay run and calculating the intra-assay coefficients of variation (%CV) for each sample/sample pool (%CV = [SD/mean] × 100%). The inter-assay variability of the ELISA was evaluated by analyzing three feline sera/serum sample pools in four consecutive assay runs and calculating the inter-assay %CVs. The possibility of high serum cobalamin concentrations affecting serum TC measurements (e.g., via binding) was tested by the paired measurement of serum TC concentrations in feline sera that were spiked with either cyanocobalamin (4 × 10^5^ ng/L; Vitamin B_12_-ratiopharm^®^ injectable solution, Ulm, Germany) or the same volume (1 μL) of assay buffer (TBS). In addition, a preliminary RI for feline serum TC concentrations was established using archived surplus serum from 41 healthy blood donor cats and the robust method after logarithmic transformation of the data (MedCalc^®^ Statistical Software v.22; https://www.medcalc.org/; accessed on 26 January 2024) [37,38].

### 2.5. Association of Serum TC with Disease and Serum Cobalamin Levels

Serum TC concentrations were measured in archived serum samples from 106 cats. This included cats with FCE (n = 28; 13 and 15 cats each with presumed or confirmed CIE or gastrointestinal neoplasia, respectively), acute enteropathy (n = 4), or other conditions (n = 74; including 24 cats with cholangiohepatopathy, 17 cats with extra-gastrointestinal neoplasia, and 33 cats with other non-neoplastic diseases). Routine diagnostics performed on these cats were at the discretion of the attending clinician and comprised a thorough patient history and physical examination. The extent and sequence of diagnostic measures were dictated by the clinical impression and overall stability of the patient. In some cats, these diagnostics included fecal parasitology, including *Giardia* spp. coproantigen test, further clinicopathologic evaluation, diagnostic imaging, and endoscopy or surgical exploration with biopsies. A diagnosis of chronic kidney disease (CKD) was based on the International Renal Interest Society (IRIS) guidelines and included persistent or progressive azotemia (increased serum phosphorus, blood urea nitrogen [BUN], symmetric dimethylarginine [SDMA], and/or creatinine concentration), decreased urine specific gravity (USG), and/or ultrasonographic findings consistent with CKD (e.g., loss corticomedullary distinction, irregular renal surface) [39].

Serum TC concentrations were compared among these groups and to those obtained in the 41 apparently healthy blood donor cats (determined based on hematology, serum biochemistry, and relevant infectious disease testing including retrovirus status). Electronic medical records were searched for all cats included in this part of the study to extract data on patient signalment, clinical, clinicopathological, and diagnostic imaging findings, and to test for their association with serum TC concentrations as well as serum cobalamin levels.

Paired serum cobalamin and TC concentrations were available from 42 cats (including 11 cats with FCE). Serum TC concentrations were also compared among groups of cats stratified by the serum cobalamin status—hypocobalaminemia defined as a serum cobalamin concentration <199 pmol/L, suboptimal serum cobalamin status (199–350 pmol/L), normocobalaminemia (350–984 pmol/L), or hypercobalaminemia (>984 pmol/L) [40].

### 2.6. Statistical Analysis

Based on the normality testing of the data using a Shapiro–Wilk *W* test, summary statistics are here reported as medians and interquartile ranges (IQR; numerical data) or counts (n) and percentages (categorical data). Non-parametric two- or multiple-group comparisons for unpaired samples were performed using a Wilcoxon rank-sum or Kruskal–Wallis test, and a Wilcoxon signed-rank test was used to compare paired data. A Spearman correlation coefficient ρ was calculated to assess for possible non-parametric correlations. Associations between categorical variables were evaluated using a likelihood ratio test (or Fisher’s exact test for *n* ≤ 5). Statistical significance was set at *p* < 0.05, and Microsoft Excel v.2410, and commercially available statistical software packages (JMP^®^ v.13, SAS Institute, Cary, NC, USA; GraphPad Prism^®^ v.10, Dotmatics, Boston, MA, USA) were used for statistical analyses.

## 3. Results

### 3.1. Antibody Specificity

**Figure 1:**
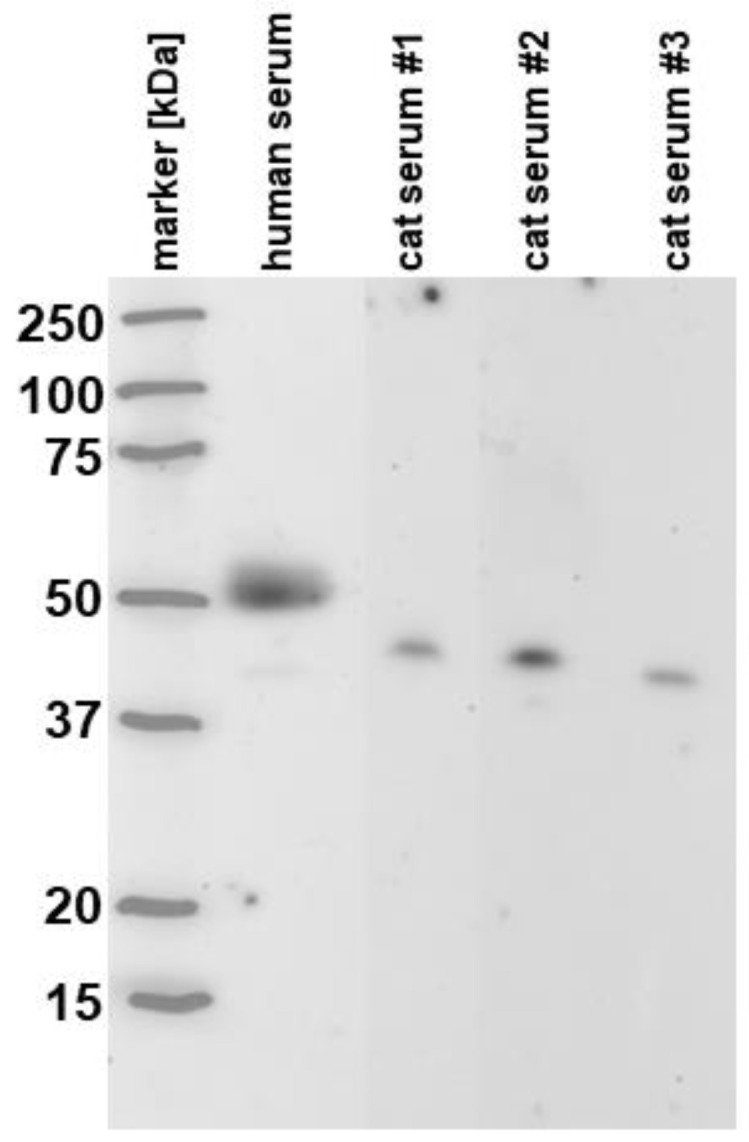
Western blot analysis to assess the specificity of the anti-transcobalamin (TC) antibodies to detect feline TC. Polyclonal anti-recombinant human TC antibodies revealed a major protein band (isoform) of TC at approximately 50 kDa and a minor band (isoform) at approximately 43 kDa in a human serum sample (lane 2). In contrast, only one band, likely the monomeric form of TC with a molecular mass of approximately 46 kDa, was detected in three different feline serum samples (lanes 3–5). Marker [kDa]: molecular weight marker lane. The original images of the Western blot are published as supplementary material.

Western blot analysis indicated a single band at a molecular mass of approximately 46 kDa in feline serum and two bands at 50 kDa (major) and 43 kDa (minor) in human serum (Figure 1).

### 3.2. ELISA Development and Validation

**Figure 2:**
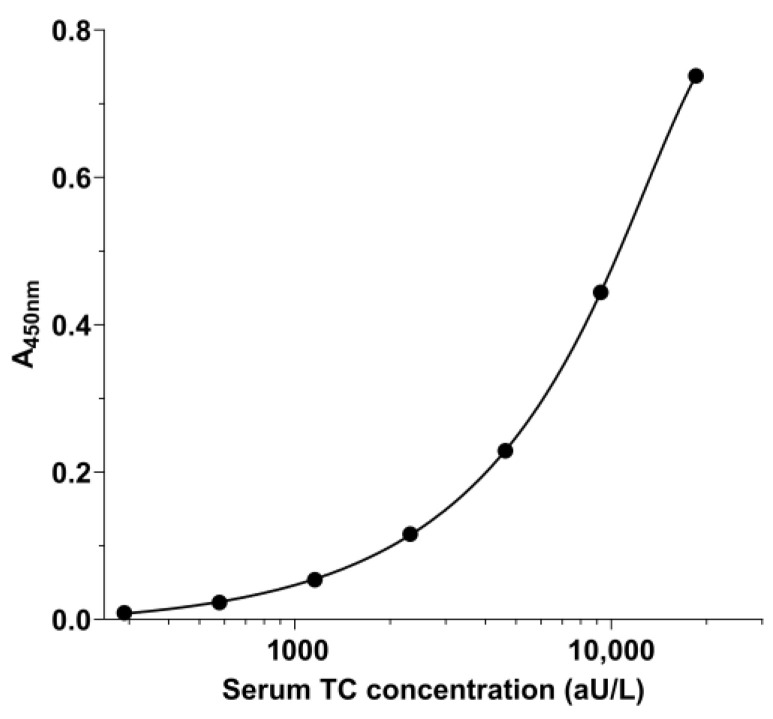
Representative calibrator curve for the sandwich-ELISA established to measure TC in feline serum. The calibrator curve was generated with a 5-parameter logistic (5PL) curve fit and using a serial dilution of human surplus serum with a known concentration of holotranscobalamin; feline serum TC concentrations (in aU/L) were extrapolated from this curve.

The sandwich-ELISA recently developed for measuring TC in serum samples from dogs [35] was successfully validated for use in feline serum samples. A representative calibration curve is shown in Figure 2.

**Figure 3:**
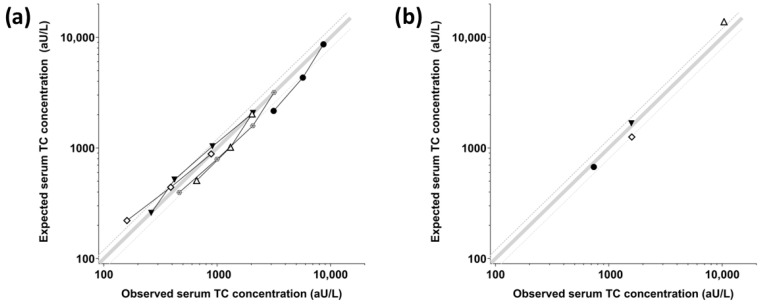
Dilutional linearity, 5 serum samples (**a**) and spike-and-recovery, 4 serum spikes (**b**) of the TC-ELISA for feline serum specimens. Observed (O) and expected (E) serum TC concentrations for (**a**) serially 2-fold diluted samples and (**b**) spiked serum samples were closely correlated, demonstrating acceptable linearity and accuracy of the TC-ELISA. Measured (observed, O) TC concentrations are plotted on the x-axis and expected (E) TC concentrations are on the y-axis. Each symbol shows a specific serum sample or serum spike, the bold gray line indicates perfect linearity or recovery (O/E ratios of 100%), and the dotted gray lines reflect the accepted range for assay dilutional linearity and spike-and-recovery (lower line, O/E ratios of 80%; upper line, O/E ratios of 120%) [36].

The LoD of the assay was determined as 80 aU/L, equating to a lower detection limit of 160 aU/L for feline serum samples tested at a 1:2 dilution. The LoQ of the assay for feline serum samples was calculated as 202 aU/L. The observed-to-expected (O/E) ratios for the serial dilutions of five feline serum samples ranged from 72.4 to 145.6%, indicating the sufficient linearity of the assay (Table 1, Figure 3a).

The spike-and-recovery O/E ratios for four spiked feline sera/sample pools ranged from 75.1 to 126.7%, demonstrating that the assay is also sufficiently accurate for feline serum samples (Table 2, Figure 3b).

The %CVs for intra- and inter-assay variation ranged from 1.8 to 17.7% and 7.3 to 17.2%, respectively (Table 3).

**Figure 4:**
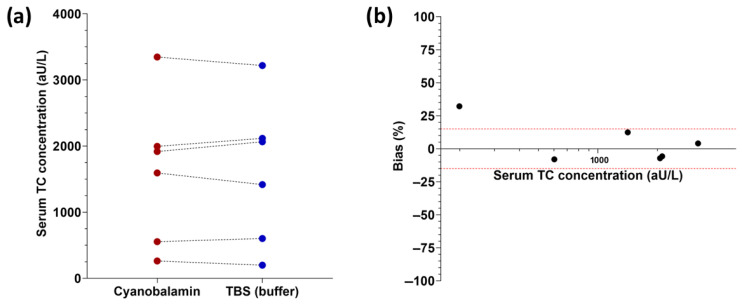
Effect of cobalamin saturation on serum TC concentrations in cats. (**a**) No significant difference was detected (*p* = 0.8438) between samples spiked with 2.95 × 10^5^ pmol/L (4 × 10^5^ ng/L) cyanocobalamin (red dots) and paired samples without cyanocobalamin added (equal volume of TBS buffer added; blue dots). (**b**) Graphical presentation as interferogram for the same samples showing that serum feline TC concentrations (x-axis) in individual samples (black dots) are affected by negligible measured bias (y-axis). The red dashed lines present the boundaries of acceptable bias (±15%).

Serum TC concentrations were not affected by spiking feline serum with cyanocobalamin for a final cobalamin concentration of 2.95 × 10^5^ pmol/L (4 × 10^5^ ng/L) vs. assay buffer prior to TC measurement (*p* = 0.8438; Figure 4a,b).

**Figure 5:**
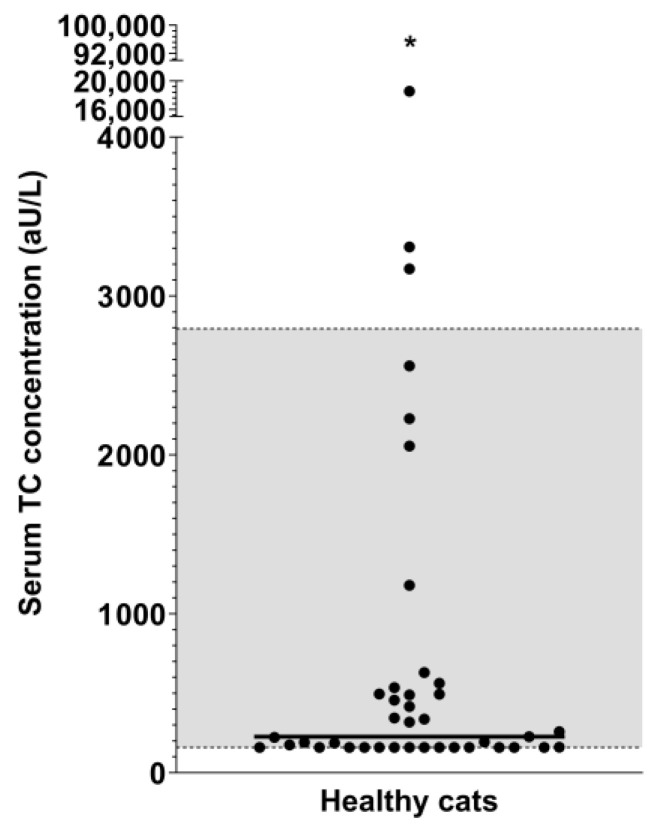
Preliminary reference interval for serum TC concentrations in cats. Serum TC concentrations ranged from 160 to 94,916 aU/L (median: 228 aU/L) in 41 healthy cats. One severe outlier (94,916 aU/L; asterisk) was excluded from the calculation of the reference interval (160–2795 aU/L; gray shaded area between dashed lines). Note the interrupted y-axis.

Serum TC concentrations in healthy cats ranged from 160 to 94,916 aU/L (median: 228 aU/L), and the RI was calculated as 160–2795 aU/L after the exclusion of one severe outlier (94,916 aU/L; Figure 5).

### 3.3. Serum TC Measurement in Clinical Cases and Association with Serum Cobalamin Level

The study included cats with FCE (*n* = 13), presumed or confirmed gastrointestinal neoplasia (GI-N; *n* = 15), cholangiohepatopathy (CH; *n* = 24), other neoplastic diseases (O-N; *n* = 17), other non-neoplastic conditions (O-N/N; *n* = 37), and healthy controls (HCo; *n* = 41). Cats in the HCo group were significantly younger and of higher body weight than cats in any of the disease groups (Table 4); no differences were seen in any other patient characteristic or clinical parameter. Serum albumin, total protein, creatinine concentrations, and ALT activities differed significantly among the groups of cats (Table 4). Serum cobalamin and folate concentrations were numerically lowest in the FCE group but were not significantly different among the disease groups of cats. Serum albumin and total protein concentrations were lowest in cats with GI-N. Cats with CH had the highest serum ALT activities and lowest serum creatinine concentrations. Serum total thyroxine (*n* = 31) and feline trypsin-like immunoreactivity (fTLI) concentrations (*n* = 1) were within the respective RI in all cats tested. Integrating serum phosphorus, BUN, SDMA, and/or creatinine concentration, USG, and ultrasonography led to a presumption of CKD in 32 cats (22%), of which 12 cats were classified as IRIS stage 1, 7 cats as IRIS stage 2, 8 cats as IRIS stage 3, and 5 cats as IRIS stage 4 [38].

**Figure 6:**
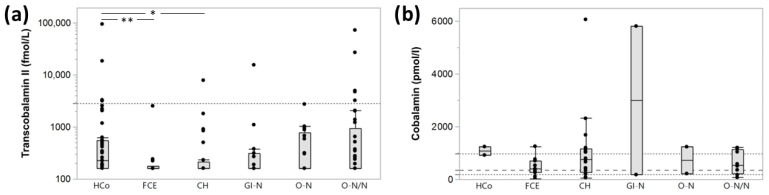
Serum TC ((**a**); n = 147) and cobalamin ((**b**); n = 42) concentrations in cats assigned to 5 major disease groups and healthy controls. Serum TC concentrations differed significantly among the different groups of cats (*p* = 0.0482), with significantly lower serum TC concentrations in cats with chronic enteropathy (FCE; *p* = 0.0067, *n* = 14) and cholangiohepatopathy (CH; *p* = 0.0153; *n* = 24) compared to healthy controls (*n* = 41). Serum cobalamin concentrations were not different among the groups of cats (*p* = 0.6340). Box plots show the median and interquartile ranges for serum TC and serum cobalamin concentrations, and the whiskers represent the inner Tukey fences; black dots represent serum TC or serum cobalamin concentrations in individual cats; the dotted lines represent the reference intervals, and the dashed line (serum cobalamin concentrations) marks the commonly recommended cut-off for cobalamin replacement therapy. * significant at *p* < 0.05; ** significant at *p* < 0.01.

Serum TC concentrations differed among the cat groups, being significantly lower in CE and CH cats than in the HCo group (Table 4, Figure 6). Post-hoc statistical power was determined as 65.6% for comparing serum TC concentrations among those groups (α = 0.05, σ = 5725, δ = 4193; mean group size, *n* = 24).

There was no difference in serum TC levels between cats with gastrointestinal lymphoma and those with lymphoma in other organs (*p* = 0.7262). Serum TC tended to correlate with serum protein concentrations, reaching significance for a moderate positive relation in HCo but showing a trend for an inverse association in GI-N cats (Table 5). A similar trend was seen for serum TC and phosphorus concentrations in O-N and between serum TC and cobalamin concentrations in FCE cats (Table 5); age and FCEAI score were not significantly related to serum TC concentrations.

**Figure 7:**
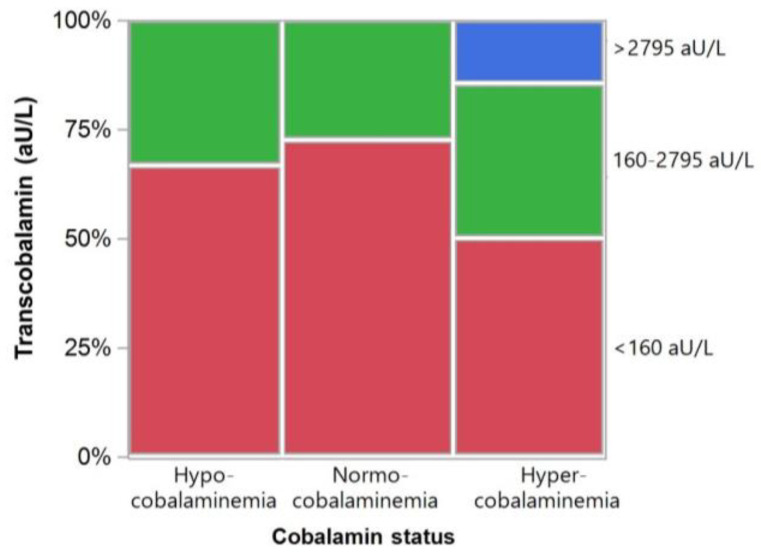
Relationship between serum TC concentrations and the serum cobalamin status in cats (*n* = 42) with paired data. Shown are the proportions of hypo-, normo-, or hypercobalaminemia cats with undetectable serum TC concentrations (<160 aU/L; red bars), detectable serum TC concentrations within the preliminary reference interval (160–2795 aU/L; green bars), or increased serum TC concentrations based on the preliminary reference interval (>2795 aU/L; blue bar).

The association between serum TC concentrations and cobalamin level did not reach statistical significance (*p* = 0.0840), but increased serum TC concentrations based on the preliminary RI (>2795 aU/L) were exclusively seen in hypercobalaminemic cats with a paired serum cobalamin measurement available from routine diagnostics (Figure 7).

The presence of CKD was associated with neither the serum concentrations of TC (*p* = 0.6255) nor cobalamin (*p* = 0.2681), and both were also not associated with the IRIS stage (both *p* < 0.05), although the highest TC concentrations were seen in cats with IRIS stages 3–4.

Cats that had received supplemental cobalamin prior to sampling had variable serum TC (160–6446 aU/L) and cobalamin concentrations (97–1262 pmol/L), which were not correlated (*p* = 0.4167).

## 4. Discussion

This is the first report on serum TC levels measured by ELISA in cats. Polyclonal antibodies raised against recombinant human TC were shown to reliably detect TC in feline serum samples, allowing for the detection and measurement of serum TC concentrations in a large number of feline specimens. This is supported by a high protein sequence homology of feline vs. human TC based on the alignment analysis (BLASTp) of 75.58% (https://blast.ncbi.nlm.nih.gov and https://uniprot.org; both accessed on 6 April 2024) and a previous immunohistochemistry study on feline neoplasms [41]. Western blot analysis further indicated that the polyclonal anti-recombinant human TC-antibodies are highly specific for the detection of TC in feline serum, demonstrating a single band in the area of the predicted molecular mass of TC (46.5 kDa). While immune cross-reactivity with close homologs (e.g., haptocorrin) cannot be entirely excluded, this appears unlikely given the low homology (33.64%) between feline TC and haptocorrin (https://blast.ncbi.nlm.nih.gov and https://uniprot.org; accessed on 27 March 2024) and the heavy glycosylation of haptocorrin (reported in humans and other species) that results in a much larger molecular mass than predicted from the protein sequence (48.1 kDa; www.ncbi.org; accessed on 2 October 2023).

A sandwich-ELISA for detecting TC in feline serum samples was successfully established by modifying a recently developed protocol for TC measurement in samples from dogs. This assay was also determined to be sufficiently linear, accurate, precise, and reproducible based on currently accepted criteria [36]. With a lower detection limit of the TC-ELISA of 160 aU/L, the ELISA yielded detectable serum TC concentrations (i.e., serum TC measurements above the LoD of the ELISA) in about 60% of all tested samples (147/254), which is consistent with the corresponding findings in dogs (64%) [35] but difficult to compare to TC concentrations (LoD 1.6 pmol/L) measured via ELISA in humans [34]. Attempts to increase the assay’s analytical sensitivity further by testing undiluted serum samples from cats (as reported for dogs [35]) were not successful due to significant matrix effects. Observed-to-expected ratios for serial dilutions and spike-and-recovery tests were mostly within the acceptable range of 70–130%, and intra- and inter-assay coefficients of variation below the accepted maximum of 15% [36], confirming that the ELISA is suitable for an initial assessment and further study of the role of TC in cobalamin metabolism and cobalamin deficiency in various disorders in cats. Still, the use of an antibody raised against feline TC might offer superior assay sensitivity. Spiking feline serum with a supraphysiological concentration of cyanocobalamin did not affect serum TC concentrations, which suggests that the ELISA measures TC independent of the serum cobalamin concentration and, thus, possible saturation with cobalamin. A preliminary RI for serum TC in cats was established (<160–2795 aU/L), which is difficult to compare to other species given the differences in assay formats and calibrators used [42,43,44]; this TC-ELISA does not distinguish between saturated (holo) and unsaturated (apo) TC [34].

This study found no significant correlation between serum TC and serum cobalamin concentrations across all groups of cats investigated, but a strong trend (*p* < 0.1) was seen for an inverse association in cats with FCE. This differs from preliminary results reported in dogs, showing significantly higher serum TC concentrations in CE dogs with suboptimal serum cobalamin levels compared to dogs with CE and either hypocobalaminemia or normo-/hypercobalaminemia [35]. Different from dogs, increased serum TC concentrations were predominantly seen in hypercobalaminemic cats for which paired analysis was available. This would suggest species-specific differences in TC-dependent cobalamin transport and metabolism. However, as a limitation, not all clinical and/or clinicopathologic parameters were available from routine diagnostics for all cats retrospectively included in the study. Further, the non-invasive assessment of health in the control group does not preclude the presence of an occult disease process (e.g., FCE) in individual cats assigned to the healthy control group [45], and serum cobalamin concentrations were also not available from all healthy control cats. Also, a more sensitive assay allowing for the detection of serum TC concentrations in all cats might have yielded more pronounced differences between groups and/or correlations with individual patient parameters.

The feline chronic enteropathy activity index (FCEAI) scores, calculated for *n* = 55 cats but not including the criterion of endoscopic lesions in most cases, did not differ significantly among the groups and had no significant correlation with serum TC concentrations. The FCEAI score does not include the evaluation of serum cobalamin as a criterion [6], but a recent study showed higher FCEAI scores to significantly correlate with lower serum cobalamin concentrations [46]. Our data suggest that serum cobalamin transport capacity (i.e., serum TC concentrations) is neither determined by the clinical disease severity nor by the serum cobalamin concentration. However, future studies will need to clarify whether the plasma cobalamin portion that is bound to TC—and is thus directly available to the tissues via the cellular TC receptor (TC-R, also known as CD320)—serves as a potential marker for cellular cobalamin availability [16,47]. The binding of cobalamin-saturated TC to the TC-R is a specific and calcium-dependent process that is followed by the internalization of this complex via endocytosis [16]. Both TC and the TC-R are then broken down within lysosomes, releasing free cobalamin, which acts as a cofactor in several cellular processes [48,49,50]. Because the availability of the TC-R at the plasma membrane is restricted to the newly synthesized receptor protein, cellular cobalamin uptake depends entirely on the de novo synthesis of the TC-R, which has been shown to be upregulated in actively proliferating cells [50] and together with TC in some feline tumors [41]. Thus, the intracellular availability of cobalamin after intestinal absorption is critically dependent on the presence of systemic transporters and functional cellular receptors.

Cobalamin in the form of 5′-deoxyadenosylcobalamin or methylcobalamin is an essential cofactor for the two intracellular enzymes methylmalonyl-CoA mutase and methionine synthase. The former catalyzes the formation of succinyl-CoA from methylmalonyl-CoA, and reduced availabilities of intracellular cobalamin can result in reduced enzyme activity and accumulation of MMA [11]. Intracellular cobalamin deficiency can also affect the activity of methionine synthase, which catalyzes the regeneration of methionine from homocysteine (HCY), which can result in increased serum HCY concentrations in dogs [11] but not in cats [25,51,52]. As a limitation of our study, serum MMA (and/or HCY) concentrations were not available to determine a possible association of serum TC concentrations with the intracellular cobalamin supply.

Some of the highest concentrations of TC were detected in cats with advanced-stage CKD. This finding agrees with studies in human medicine showing holoTC to increase in renal patients [53,54], and could be due to decreased TC glomerular filtration, which results in reduced clearance and prolonged plasma half-life. Another possible explanation could be that there is resistance to the vitamin [55] or a compensatory response to the reduced proximal tubular reabsorption of filtered cobalamin [56]. CKD is known to be a common clinical problem in elderly patients [57]. However, despite age being significantly different between the groups of cats in this study, we did not document a significant correlation of age with serum TC concentrations. Thus, a further evaluation of cobalamin metabolism and the role of TC in feline CKD is warranted.

This study found a significant relationship between serum TC and serum total protein concentrations in the healthy control group and a similar trend in the group of cats with gastrointestinal neoplasia. While there are currently no reliable data to suggest that TC presents either a positive or negative acute-phase reactant in humans [34,58], this response may be species-specific, as shown for other acute-phase molecules such as serum C-reactive protein (CRP; particularly in dogs), serum amyloid A (SAA; particularly in cats), or serum alpha_1_-antitrypsin [59,60]. In addition, there is evidence that serum TC concentrations can also be extremely highly increased with certain systemic or neoplastic diseases such as proliferative (reactive or malignant) histiocytosis, which is characterized by the increased activity of the monocyte/macrophage system [34,41,61].

Treatment-naïve hyperthyroidism can lead to transient hypocobalaminemia in cats [27], and several possible causes have been proposed for this self-limiting change. On the one hand, it is suspected that structural and/or functional ileal or pancreatic compromise might play a role in affected cats. However, an accelerated metabolism with increased protein turnover and increased demand for cobalamin due to uncontrolled hyperthyroidism could also contribute to hypocobalaminemia, as could an increased renal excretion of the vitamin and/or its binding proteins [26,27,62]. Interestingly, two of the cats in this study with concurrent confirmed (increased serum total T4 concentration) or presumed (high-normal serum T4 concentration) hyperthyroidism had serum TC levels (<160 aU/L and 305 aU/L) that would be consistent with several of these possible explanations.

This study confirmed that acute gastroenteritis, which might occur concurrently with pancreatic and biliary tract disease, can also cause cobalamin deficiency [52] and an increase in serum TC concentration, which likely reflects a compensatory (i.e., physiological) response. In addition to a functional deficiency, an abnormal presence of cobalamin-binding bacteria (e.g., *Clostridium* spp.) is assumed to lead to a reduced bioavailability of cobalamin [11,12]. Some bacteria, such as the Gram-negative anaerobes of the genus *Bacteroides*, are also able to interfere with mechanisms of cobalamin metabolism, such as the binding of IF [63].

## 5. Conclusions

The TC-ELISA was shown to be sufficiently analytically sensitive, linear, accurate, precise, and reproducible for TC detection in feline samples. This assay will allow further study of the role of TC in cobalamin metabolism in FCE and other conditions in cats, but a species-specific ELISA might offer superior sensitivity. TC variation associated with cobalamin deficiency states in FCE (similar to cases of cholangiohepatopathy) could result from the inflammatory response or anti-TC autoantibodies, as described in humans. Further studies are warranted to determine whether serum TC variation in cats with FCE contributes to reduced intracellular availability of cobalamin, changes with supplementation, and is affected (and to what extent) by concurrent CKD.

## Figures and Tables

**Table 1:** Dilutional linearity of serum samples from cats using the sandwich TC-ELISA. The observed-to-expected ratios for the serial two-fold dilution of five different serum samples ranged from 72.4 to 145.6% (mean ±SD: 111.6 ± 23.2%).

Sample	Dilutions	Serum TC (aU/L)	Observed/ Expected ± SD (%)
1	1:2–1:8	768	80.2 ± 7.8
2	1:2–1:8	2429	129.3 ± 0.5
3	1:2–1:8	10,886	138.6 ± 7.0
4	1:2–1:16	1914	90.0 ± 8.3
5	1:2–1:16	3747	124.2 ± 5.5

SD: standard deviation.

**Table 2:** Spike-and-recovery of TC in serum samples measured by the sandwich-ELISA. Observed-to-expected ratios for four serum spikes obtained by mixing eight different serum samples or sample pools ranged from 75.1 to 126.7% (101.4 ± 19.0%).

Spike Specimen	Serum 1 TC (aU/L)	Serum 2 TC (aU/L)	Observed Serum TC (aU/L)	Expected Serum TC (aU/L)	Observed/ Expected (%)
1	1244	103	736	674	109.3
2	1705	811	1594	1258	126.7
3	2072	1270	1580	1671	94.5
4	632	27,032	10,381	13,832	75.1

**Table 3:** Intra- and inter-assay variation of the ELISA for TC measurement in feline serum samples.

Serum Sample		Mean ± SD (aU/L)	CV (%)		Serum Sample		Mean ± SD (aU/L)	CV (%)
	1	432 ± 47	10.8			1	510 ± 88	17.2
Intra-assay	2	684 ± 26	3.9	Inter-assay		2	4947 ± 499	10.1
Variation	3	2457 ± 434	17.7	Variation		3	5699 ± 416	7.3
	4	5899 ± 105	1.8					

CV, coefficient of variation; SD, standard deviation.

**Table 4:** Patient characteristics, clinical findings, and clinicopathologic parameters in cats (n = 147) with chronic inflammatory enteropathy (FCE), presumed or confirmed gastrointestinal neoplasia (GI-N), cholangiohepatopathy (CH), other neoplastic disease (O-N), other non-neoplastic conditions (O-N/N), and healthy controls (HCo).

Parameter	FCE	GI-N	CH	O-N	O-N/N	HCo	*p*-Value
N	13 *	15	24	17	37	41	–
Patient characteristics							
Age, in years	**11** ^A^ (4–12.5)	**10** ^A^ (4–11)	**8.5** ^A,B^ (2–11)	**9** ^A^ (6–11.5)	**8** ^A^ (3–10.5)	**4** ^B^ (2–6.5)	**0.0014**
Sex,							0.7038
male (neutered)/female (spayed)	8 (7)/ 5 (5)	8 (8)/ 7 (6)	12 (11)/ 12 (11)	12 (9)/ 5 (5)	25 (22)/ 12 (8)	26 (19)/ 15 (11)	
Body weight, in kg ^†^	**4.3** ^A,B^ (4.0–5.0)	**3.6** ^A^ (3.0–4.6)	**3.9** ^A,B^ (2.9–5.6)	**4.5** ^B,C^ (3.8–5.6)	**4.5** ^B,C^ (3.3–6.3)	**5.1** ^C^ (4.4–5.8)	**0.0209**
Breed							0.3916
Domestic (European) Shorthair	8 (62%)	12 (80%)	16 (67%)	13 (76%)	24 (65%)	34 (83%)	
Other breeds	5 (38%)	3 (20%)	8 (33%)	4 (24%)	13 (35%)	7 (17%)	
FeLV–status ^$^	0/7 (0%)	0/7 (0%)	1/16 (6%)	1/10 (10%)	1/11 (9%)	0/35 (0%)	–
FIV–status ^$^	0/7 (0%)	0/7 (0%)	0/16 (0%)	1/10 (10%)	2/11 (18%)	0/25 (0%)	
Clinical parameters							
Clinical signs							–
vomiting	9/13 (69%)	7/15 (47%)	11/24 (46%)	3/17 (18%)	17/37 (46%)	0/41 (0%)	
diarrhea	5/13 (20%)	3/15 (20%)	5/24 (21%)	2/17 (12%)	7/37 (19%)	0/41 (0%)	
hypo-/anorexia	8/13 (62%)	11/15 (73%)	16/24 (67%)	13/17 (77%)	21/37 (57%)	0/41 (0%)	
weight loss	3/13 (23%)	10/15 (67%)	12/24 (50%)	8/17 (47%)	8/37 (22%)	0/41 (0%)	
lethargy	7/13 (53%)	8/15 (53%)	17/24 (71%)	11/17 (65%)	21/37 (57%)	1/41 (2%)	
FCEAI score ^¶^	8.5 (5–9.5)	6 (5–9)	7 (6–9)	7 (5–9)	9 (3.5–15)	–	0.7699
Clinicopathologic serum parameters							
Cobalamin, in pmol/L ^‡^	404 (262–705)	3000 (179–5821)	748 (282–1152)	729 (220–1238)	548 (221–1140)	1083 (922–1244)	0.6340
Hypocobalaminemia	2/11 (18%)	0/2 (0%)	2/17 (12%)	0/2 (0%)	2/8 (25%)	0/2 (0%)	0.0869
Hypercobalaminemia	1/11 (9%)	1/2 (50%)	7/17 (41%)	1/2 (50%)	3/8 (38%)	1/2 (50%)	
Folate, in nmol/L ^$^	29.9 (25.8–35.1)	44.3	31.2 (24.1–47.4)	–	30.6 (25.3–31.9)	–	0.5801
Hypofolatemia ^$^	2/9 (22%)	0/1 (0%)	2/7 (29%)	–	1/6 (17%)	–	0.8395
Hyperfolatemia	1/9 (11%)	0/1 (0%)	0/7 (0%)		0/6 (0%)		
Albumin, in g/L **	**31** ^A,B^ (28–32)	**24.5** ^C^ (23–27)	**27** ^A,C^ (23–32)	**31** ^A,C,D^ (25–36)	**32** ^B,D^ (30–34)	**32.5** ^D^ (31–36)	**<0.0001**
Globulin, in g/L **	38 (33–48)	38.5 (37–42)	39.5 (30–43)	38 (32–49)	43 (36–49)	41 (39–45)	0.1214
Total protein, in g/L **	**69** ^A,B,C^ (61.5–77)	**63.5** ^A,C^ (61–72.5)	**66.5** ^A,C^ (58–76.5)	**74** ^A,B,C,D^ (64–76)	**74.5** ^B,D^ (69–82)	**74.5** ^D^ (72–79)	**0.0002**
Hyperproteinemia	3/14 (21%)	1/14 (7%)	4/24 (17%)	2/15 (13%)	14/36 (39%)	12/38 (32%)	0.0896
ALT, in U/L ^††^	**55.5** ^A,B,C^ (36–75)	**32** ^A^ (26.5–62)	**161.5** ^B^ (43–363)	**39** ^A^ (28–57)	**58** ^C^ (38–95)	**51** ^C^ (44.5–65.5)	**0.0003**
Increased ALT ^††^	3/14 (21%)	2/13 (15%)	15/24 (63%)	3/15 (20%)	10/35 (29%)	1/37 (3%)	**<0.0001**
ALP, in U/L ^††^	26 (20–48)	40 (21.5–63.5)	47.5 (25–129)	29 (19–36)	34 (26–62)	34 (25–41)	0.1581
Increased ALP ^††^	3/14 (21%)	4/13 (31%)	11/24 (46%)	3/15 (20%)	9/34 (27%)	5/38 (13%)	0.0556
γGT, in U/L ^$$^	3.5 (1–5)	4 (1–5.5)	4 (2–8)	1 (1–2.5)	2 (1–3)	3 (1–4)	0.0719
Phosphorus, in mmol/L ^‡‡^	1.4 (1.2–2.1)	1.4 (1.3–3.9)	1.4 (0.9–1.6)	1.4 (1.2–1.7)	1.9 (1.5–2.9)	1.4 (1.1–1.6)	0.0870
Hyperphosphatemia ^‡‡^	3/9 (33%)	3/10 (30%)	2/20 (10%)	1/12 (8%)	8/18 (44%)	0/3 (0%)	0.0644
Creatinine, in μmol/L ^††^	**131** ^A^ (108–167)	**125** ^A,B^ (77–205)	**88** ^B^ (67–105)	**117** ^A^ (97–135)	**122** ^A^ (77–197)	**119** ^A^ (105–131)	**0.0068**
BUN, in mmol/L ^§§^	8.8 (6.1–11.6)	8.6 (6.0–22.1)	8.5 (5.7–11.0)	10.2 (6.0–17.3)	11.2 (6.8–19.3)	9.1 (8.0–10.0)	0.2048
Spec fPL, in μg/L ^¶¶^	1.4 (0.7–3.2)	6.6 (2.9–10.3)	2.5 (1.7–10.4)	–	1.4 (1.0–5.1)	4.5 (1.0–8.0)	0.1558
Transcobalamin II, in aU/L, median (IQR)	**160** ^A^ (160–178)	**160** ^A,B^ (160–314)	**160** ^A^ (160–214)	**160** ^A,B^ (160–781)	**160** ^A,B^ (160–939)	**160** ^B^ (160–551)	**0.0482**

FCEAI: feline chronic enteropathy activity index. IQR: interquartile range. Spec fPL: specific pancreatic lipase. Numerical values are presented as medians (IQR), and n (%) are reported for categorical data. Parameters in bold font and differential superscript numbers (A, B, C, or D) indicate significant differences at *p* < 0.05 (bold *p* values). * includes 14 serum samples (one cat sampled twice 9 months apart); ^†^ available from n = 143 cats; ^$^ available from n = 86 cats; ^‡^ available from n = 42 cats; ^¶^ calculated for n = 55 cats (11 cats with FCE); ** available from n = 141 cats; ^††^ available from n = 138 cats; ^$$^ available from n = 65 cats; ^‡‡^ available from n = 72 cats; ^§§^ available from n = 139 cats; ^¶¶^ available from n = 34 cats.

**Table 5:** Correlation among serum transcobalamin II (TC) concentrations and patient characteristics, clinical, and laboratory findings in all cats included in the study (*n* = 147) and stratified into disease groups (FCE, GI-N, CH, O-N, O-N/N) or healthy controls (HCo). The relationships between serum TC concentrations, age, feline chronic enteropathy activity index (FCEAI) score, and clinicopathologic results (serum albumin, globulin, total protein, cobalamin, and folate concentration) are summarized.

Serum TC		Spearman ρ Correlation Coefficient (*p*)						
	Correlated with	All Cats	FCE	GI-N	CH	O-N	O-N/N	HCo
**Patient characteristics**								
Age		0.04 (0.6627)	−0.20 (0.4850)	0.31 (0.2559)	0.14 (0.5051)	−0.21 (0.4246)	0.23 (0.1770)	0.20 (0.2101)
Clinical criteria								
FCEAI score		−0.16 (0.2455)	−0.18 (0.6023)	0.03 (0.9158)	−0.27 (0.2089)	n/a	−0.22 (0.7177)	n/a
Serum protein concentrations								
Albumin concentration		0.09 (0.2714)	0.27 (0.3443)	0.43 (0.1228)	0.20 (0.3430)	0.01 (0.9893)	−0.05 (0.7733)	0.04 (0.8300)
Globulin concentration		0.11 (0.1776)	0.15 (0.6158)	−0.29 (0.3118)	−0.09 (0.6923)	−0.07 (0.8151)	0.05 (0.7727)	0.29 (0.0730)
Total protein concentration		0.16 (0.0564)	0.08 (0.7948)	−0.52 (0.0555)	0.04 (0.8505)	0.05 (0.8620)	0.06 (0.7403)	0.41 (0.0102)
Serum renal markers								
Creatinine concentration		0.01 (0.8900)	−0.03 (0.9256)	0.02 (0.9605)	0.25 (0.2690)	−0.16 (0.5570)	0.04 (0.8122)	−0.20 (0.2437)
BUN concentration		0.06 (0.4764)	0.13 (0.6693)	−0.15 (0.6184)	0.08 (0.7287)	−0.37 (0.1594)	0.18 (0.3020)	0.28 (0.0879)
Phosphorus concentration		−0.13 (0.2735)	−0.55 (0.1269)	−0.52 (0.1203)	0.07 (0.7675)	−0.54 (0.0711)	−0.03 (0.9204)	−0.87 (0.3333)
Serum functional biomarker								
Cobalamin concentration		−0.01 (0.9484)	−0.54 (0.0840)	n/a	−0.07 (0.7929)	n/a	0.51 (0.1922)	n/a
Folate concentration		0.11 (0.6337)	0.10 (0.7999)	n/a	−0.08 (0.8666)	n/a	−0.03 (0.9493)	n/a

BUN: blood urea nitrogen. n/a: not applicable. Green-shaded cells indicate a significant (*p* < 0.05) correlation, whereas red-shaded cells reflect trends (*p* < 0.1) for a correlation.

## Data Availability

Data (anonymized) are made available from the first or last author upon reasonable request.

## Supplementary Materials

The following is available at https://www.mdpi.com/article/10.3390/vetsci11110552/s1, Figure S1: Original image of the Western blot analysis (left panel) and cropped image (right panel) to assess the specificity of the polyclonal anti-recombinant human transcobalamin (TC) antibodies to detect feline TC.
