# Supplementary figures and images for: Serum Transcobalamin Concentration in Cats—Method Validation and Evaluation in Chronic Enteropathies and Other Conditions
Source: Vet Sci. 2024 Nov 9;11(11):552. doi: 10.3390/vetsci11110552 (PMC11599117; doi:10.3390/vetsci11110552)

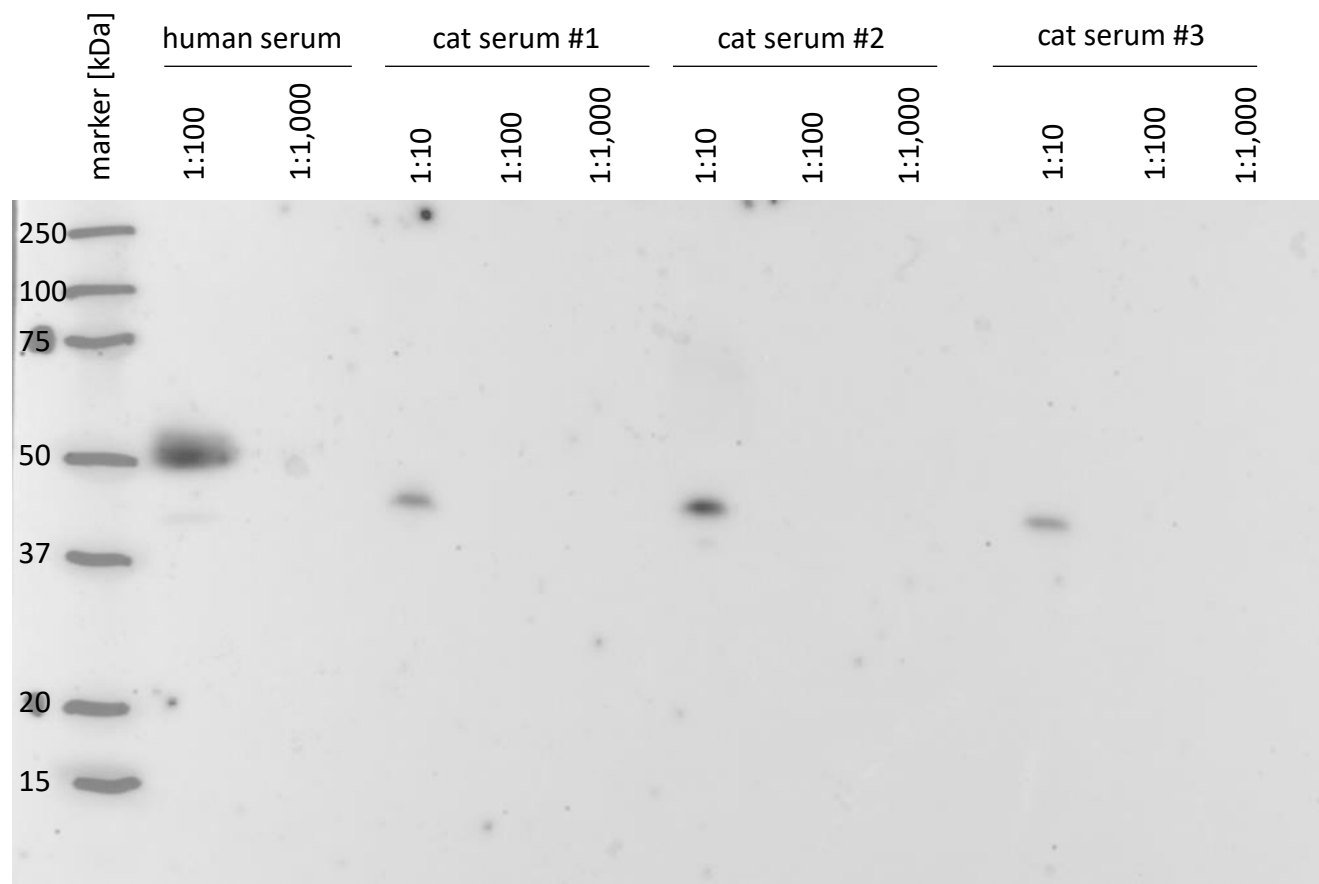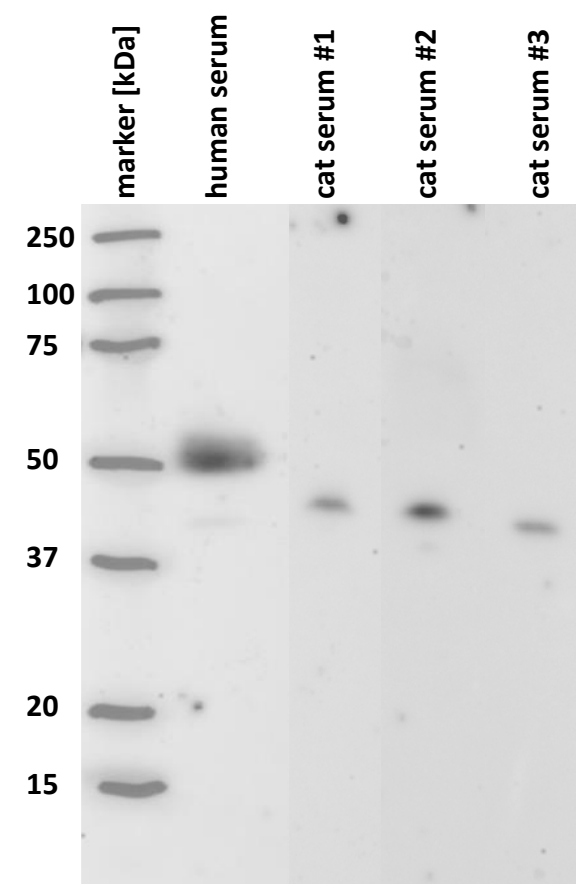

Supplement: Supplementary file 1 [file vetsci-11-00552-s001.zip › vetsci-3242172-supplementary.pdf]
